# Validation of the application of gel beads-based single-cell genome sequencing platform to soil and seawater

**DOI:** 10.1038/s43705-022-00179-4

**Published:** 2022-09-29

**Authors:** Yohei Nishikawa, Masato Kogawa, Masahito Hosokawa, Ryota Wagatsuma, Katsuhiko Mineta, Kai Takahashi, Keigo Ide, Kei Yura, Hayedeh Behzad, Takashi Gojobori, Haruko Takeyama

**Affiliations:** 1 Research Organization for Nano & Life Innovation, Waseda University, 513 Waseda tsurumaki-cho, Shinjuku-ku, Tokyo 162–0041 Japan; 2 Computational Bio Big-Data Open Innovation Laboratory, AIST-Waseda University, 3-4-1 Okubo, Shinjuku-ku, Tokyo 169-8555 Japan; 3 Department of Life Science and Medical Bioscience, Waseda University, 2-2 Wakamatsu-cho, Shinjuku-ku, Tokyo 162–8480 Japan; 4 Institute for Advanced Research of Biosystem Dynamics, Waseda Research Institute for Science and Engineering, Graduate School of Advanced Science and Engineering, Waseda University, 3-4-1 Okubo, Shinjuku-ku, Tokyo 169-8555 Japan; 5 Computer, Electrical and Mathematical Sciences and Engineering (CEMSE) Division, King Abdullah University of Science and Technology (KAUST), Thuwal, 23955-6900 Saudi Arabia; 6 Computational Bioscience Research Center (CBRC), King Abdullah University of Science and Technology (KAUST), Thuwal, 23955-6900 Saudi Arabia; 7 Graduate School of Humanities and Sciences, Ochanomizu University, 2-1-1 Otsuka, Bunkyo-ku, Tokyo 112-8610 Japan; 8 Biological and Environmental Sciences and Engineering (BESE) Division, King Abdullah University of Science and Technology (KAUST), Thuwal, 23955-6900 Saudi Arabia

**Keywords:** Environmental microbiology, Next-generation sequencing

## Abstract

Single-cell genomics is applied to environmental samples as a method to solve the problems of current metagenomics. However, in the fluorescence-activated cell sorting-based cell isolation and subsequent whole genome amplification, the sorting efficiency and the sequence quality are greatly affected by the type of target environment, limiting its adaptability. Here, we developed an improved single-cell genomics platform, named SAG-gel, which utilizes gel beads for single-cell isolation, lysis, and whole genome amplification. To validate the versatility of SAG-gel, single-cell genome sequencing was performed with model bacteria and microbial samples collected from eight environmental sites, including soil and seawater. Gel beads enabled multiple lysis treatments. The genome coverage with model bacteria was improved by 9.1–25%. A total of 734 single amplified genomes were collected from the diverse environmental samples, and almost full-length 16S rRNA genes were recovered from 57.8% of them. We also revealed two marine *Rhodobacter* strains harboring nearly identical 16S rRNA genes but having different genome contents. In addition, searching for viral sequences elucidated the virus-host linkage over the sampling sites, revealing the geographic distribution and diverse host range of viruses.

## Introduction

Technological innovations in metagenome sequencing have improved our understanding of the genomic characteristics, genetic diversity, and metabolic capabilities of microbial communities in diverse environments [1, 2]. The advances in computational techniques, such as metagenome binning, have enabled the generation of metagenome-assembled genomes (MAGs) [3, 4]. Metagenome binning has been applied to metagenomic sequencing data obtained from various environments, providing an expedient path for exploring microbial dark matter. However, current metagenomic binning approaches have several limitations in terms of genome reconstruction, including reconstruction of massive MAGs from complex microbial communities such as those in soil and sediment [5, 6]; recovery of 16S rRNA genes using MAGs [7]; the distinction of closely related genomes [8]; and detection of viral sequences to predict the host range of viruses [9].

Single-cell genome sequencing is expected to be an alternative approach to resolve these problems [10, 11]. In contrast to metagenomic binning approaches, single amplified genomes (SAGs) can provide sequence information on individual cells, allowing the identification of heterogeneity within a bacterial community, including closely related species [12]. So far, single-cell resolution sequence data have enabled the detection of plasmids [13] and the analysis of virus-host interactions [14, 15]. In particular, droplet microfluidics is considered a noteworthy technique and as a high-throughput single-cell analysis tool [16–18]. However, in the conventional droplet-based single-cell genomics, the immiscible oil phase makes it difficult to conduct multistep reactions, causing an obstacle in the lysis of gram-positive and -negative bacteria and in the exclusion of inhibitors for whole genome amplification (WGA). Sample preparation is also a critical step, and current single-cell genomics faces difficulty in the analysis of soil samples containing tiny particles, reducing the accuracy and throughput of WGA [19].

In this study, we propose an improved protocol of agarose gel beads-based multiplexed single-cell genome sequencing, named single-cell amplified genomes in gel beads (SAG-gel) [20], as an alternative to current metagenomics and single-cell genomics. Gel beads generated by a microfluidic device enabled high-throughput WGA of more than 10^5^ cells at a time in a single tube. In addition, validation with model bacteria revealed that SAG-gel improves the sequence quality compared to conventional single-cell genome sequencing methods by combining multiple cell lysis steps. To demonstrate the versatility of SAG-gel, we conducted extensive single-cell genomics with eight environmental samples, including those from soil and seawater. The diverse environmental samples, ranging from marine to terrestrial, were collected from the Red Sea as well as from nearby mangrove forests and deserts. All the sampling sites are exposed to harsh conditions such as high temperatures, intensive ultraviolet light, and low nutrients throughout the year, and are recognized as one of the attractive target sites for microbial analysis [21]. A total of 735 SAGs across 231 species were obtained, 98.7% of which have not been previously described in the database. SAG-gel showed a higher recovery rate of 16S rRNA genes than did conventional methods, which enabled strain-level comparative genomics of SAGs with high 16S rRNA gene identity. Furthermore, SAG-gel enabled the detection of characteristic genes, such as biosynthetic gene clusters (BGCs) and viral sequences in individual SAGs, revealing differences in their metabolic functions and traces of viral infections at the single-cell level.

## Results

### Single-cell genome sequencing of model bacteria

**Fig. 1:**
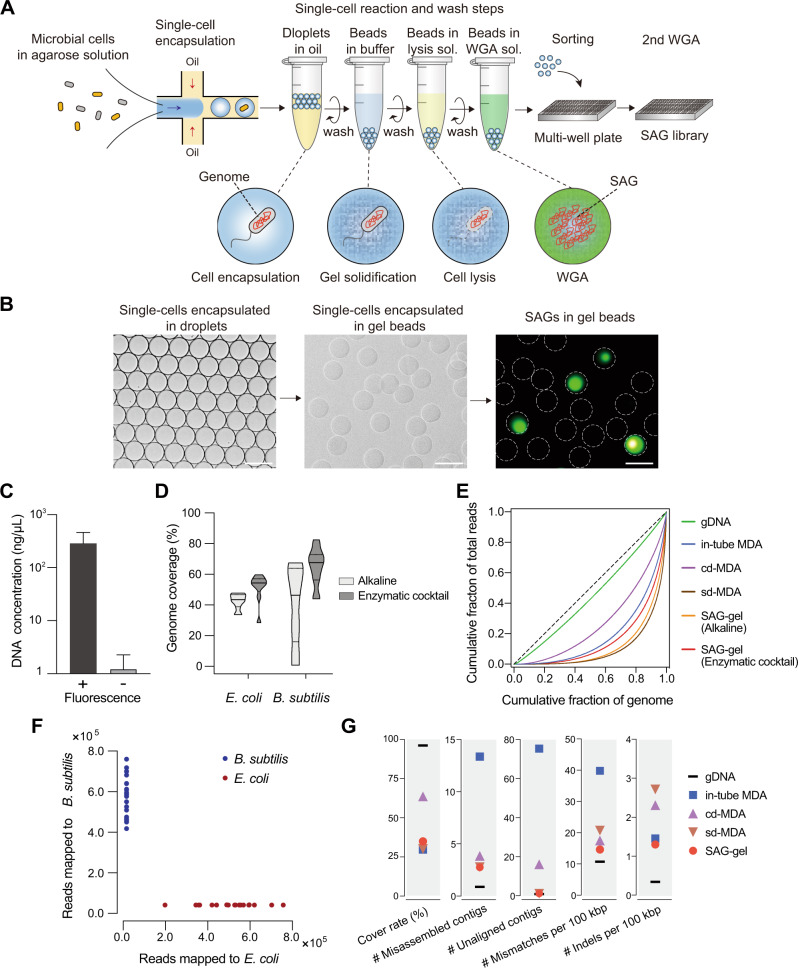
Single-cell amplified genome in the gel beads (SAG-gel). **A** Bacterial suspensions are encapsulated into 40 μm of microfluidic droplets at the single-cell level (0.1 cell/droplet) with ultra-low melting temperature agarose. After solidification, gel-beads are transitioned into the aqueous phase, and proceed to cells lysis and whole genome amplification (WGA). Gel-beads after WGA are isolated with FACS and proceed to library preparation and next generation sequencing. **B** Microscopic image of microfluidic water in oil (W/O) droplets and gel-beads. Amplified DNA can be visualized by adding DNA intercalating dye (SYBR Green). The scale bar is 50 μm. **C** DNA concentration after 2nd-round WGA of fluorescence-positive and -negative beads (**D**) Comparison of genome coverage of *E. coli* and *B. subtilis* treated with alkaline or enzymatic cocktail. Genome coverage was evaluated at the ×10 sequence depth. **E** Lorenz curve for evaluation of amplification biases in *E. coli* single-cell sequence data (**F**) The scatter plot shows the number of reads mapped to *E. coli* and *B. subtilis* genomes associated with each droplet. **G** Sequence statistics of contigs obtained from SAGs of *E. coli*. Of the five methods compared, all data except the genomic DNA (gDNA) data, were obtained using single-cell genome sequencing methods, including compartmented droplet MDA (cd-MDA) [16] and single-droplet MDA (sd-MDA) [17].

The SAG-gel strategy involves performing a series of reactions on encapsulated single cells in a massively parallel manner (Fig. 1A). Single cells are massively captured in gel beads with a microfluidic device [17] and then lysed by enzyme cocktails for both gram-positive and -negative bacteria, which is modified from our previous method [20] (See Supplementary Information for details). After 1st-round WGA, SAG-gel amplifies the genomes of various bacterial cells within uniform-shaped beads floating in a single tube (Fig. 1B). The gel matrix facilitates maintenance of the compartmented genomes during cell lysis, washing, and WGA. The fluorescence-positive beads are isolated into multi-well plates with FACS and processed for the subsequent 2nd-round WGA, library preparation, and next generation sequencing. In the validation study with model bacteria (*Escherichia coli* and *Bacillus subtilis*), >95% of fluorescence positive-sorted gel beads exhibited sufficient DNA amplification (>1.2 μg) after the 2nd-round WGA, while negative-sorted gel beads did not show obvious background amplification (<2.8 ng) (*n* = 5) (Fig. 1C and Table S1). The enzyme cocktails enhanced the genome coverage by 9.1–25% and improved genome amplification biases compared to conventional alkaline lysis treatment (Fig. 1D, E). We confirmed that SAG-gel eliminates the risk of cross-contamination between gel beads, with all sequenced SAGs having >99.6% of their reads mapped to either *E. coli* or *B. subtilis* (Fig. 1F). The de novo assembled contigs showed a lower number of misassembled and unaligned contigs, a longer length of N50 (9698 bp), and a lower number of mismatches (13.5 per 100 kbp), and indels (1.00 per 100 kbp) in SAG-gel than in conventional in-tube WGA reactions (Fig. 1G and Table S2).

### Single-cell genome sequencing of eight environmental samples, including soil and seawater

**Fig. 2:**
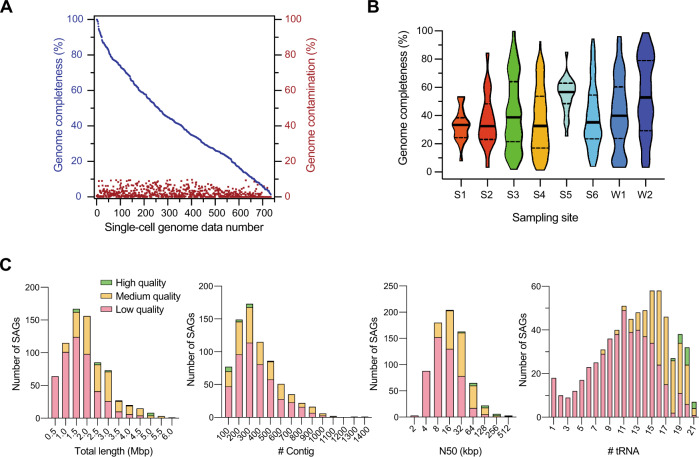
Sequence statistics for 734 SAGs collected from eight environmental samples. **A** Genome completeness and contamination of 734 SAGs obtained from eight environmental sites. **B** Distributions of SAG completeness for the eight environmental samples including six soil samples (beach soil: S1; desert soil: S2; mangrove soil: S3; fresh sea sediment: S4; frozen sea sediment: S5; and seashore soil: S6) and two seawater samples (harbor seawater: W1; and open-ocean seawater: W2). Solid line is the median and the dotted line is the quartile. **C** Distributions of the total length, the number of contigs, N50, and the number of tRNA classified by the quality of SAGs.

We applied SAG-gel to eight environmental samples including six soil samples (beach soil: S1; desert soil: S2; mangrove soil: S3; fresh sea sediment: S4; frozen sea sediment: S5; and seashore soil: S6) and two seawater samples (harbor seawater: W1; and open-ocean seawater: W2). Details of the sampling, preparation of microbial fractions, and the process of SAG-gel are provided in the Supplementary Information. After preparing cell suspensions, cells were encapsulated in microfluidic droplets at 0.1 cell/droplet and then proceeded to 1st-round WGA in gel beads. The rate of fluorescence-positive gel beads was 13.2% as the highest, confirming that the gel beads encapsulating multiple cells were theoretically <1%. We individually generated 1970 SAG reactions from isolated gel beads, of which 1031 were identified as positive bacterial and archaeal fractions by 16S rRNA gene PCR (Table S3). When analyzing model bacteria, negative-sorted gel beads showed no obvious amplification. Sequencing and data curation from 929 samples resulted in 734 SAGs (Table S4). Using the Genomic Standards Consortium guidelines [22], 16 high-quality and 244 medium-quality SAGs were classified. Draft genomes ranged from 0.49 to 5.9 Mbp in total length and consisted of 3–1347 contigs. The average genome completeness for each sampling site was 32.9% for S1, 37.1% for S2, 42.3% for S3, 36.2% for S4, 54.8% for S5, 39.4% for S6, 42.2% for W1, and 53.6% for W2. The completeness exhibited a broad distribution between 1.37–99.9% (Fig. 2A, B). High-quality SAGs yielded an average N50 of 83.2 kb, while medium- and low-quality ones yielded 27.5 kb and 12.3 kb, respectively. Regarding the presence of tRNAs in high-to-low quality SAGs, the average numbers were 19.8, 16.5, and 10.6, respectively (Fig. 2C). Gel beads isolation combined with FACS effectively reduced the rate of contaminating SAGs (7.62% (56/735)) compared to that by isolation using manual picking (38.7% (75/194)) (Fig. S1).

### Newly identified bacterial taxonomic distribution

**Fig. 3:**
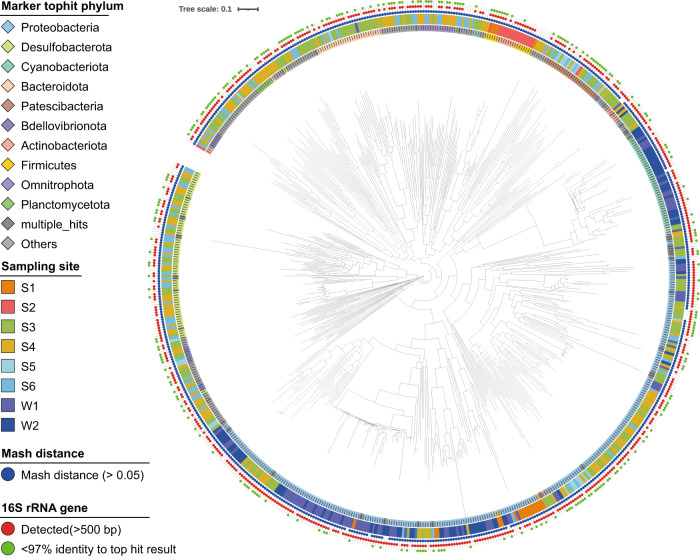
Taxonomic classifications of SAGs collected from environmental samples. The GTDB-Tk phylogenetic tree showing the taxon of 639 bacterial SAGs at the phylum level. Inner color bar: Top hit phylum; outer color bar: sampling site; blue dot: >0.05 Mash distance to the closest genomes in Refseq; red dot: 16S rRNA gene detected; green dot: <97% identity of 16S rRNA sequence to the top hit result. Any phyla that were not included in the top ten phyla were clustered as “others”.

Of the 734 SAGs, 76.0% (558/734) and 58.7% (431/734) had 16S rRNA gene sequences that were >500 bp and >1400 bp in length, respectively (Table S4). The SAG-gel platform yielded longer sequences for genome assemblies and notably higher recovery rates of 16S rRNA gene than did metagenome-based analysis (7–17%) [7, 23] and other single-cell genome analyses (23–27%) [24, 25]. The 16S rRNA gene-based phylogenetic analysis revealed that 117 (102 specific) sequences with lengths >1400 bp and 204 (173 specific) sequences lengths >500 bp had no obvious reference sequences (<97% identity to the top hit result). Taxonomic annotation resulted in 650 SAGs classified into 11 archaea and 639 bacteria, consisting of 44 phyla, 75 classes, 117 orders, and 127 families. *Proteobacteria* was the dominant [277 (43.3%)] followed by *Desulfobacterota* [70 (10.9%)] and *Cyanobacteriota* [44 (6.9%)]. We found a total of 231 species from the 650 SAGs, 98.7% (228 species) of which were newly identified with >0.05 Mash distance to reference genomes registered in Refseq [26] (Fig. 3 and Table S4). Comparison of the phylum-level taxonomic compositions of SAGs with metagenomic 16S rRNA gene amplicon sequencing showed that most of the major phyla were recovered, although bacterial composition was different between the two methods (Fig. S2). SAG-gel recovered rare phyla with <0.1% in the 16S rRNA gene sequencing (See Supplementary Information).

### Whole-genome comparative analysis of marine bacterial species with identical 16S rRNA gene

From harbor seawater samples (W1), we found 28 SAGs that shared 99.9% identity with over 1457 bp of the 16S rRNA gene. These were assigned as *Rhodobacter* spp., a common genus in freshwater or marine environments with a wide range of metabolic capabilities [27]. Results of average nucleotide identity (ANI) suggested that the 28 SAGs fell into several clusters, including two large clusters (*Rhodobacter* spp. RS1 and RS2) (Fig. S3A). We then combined these SAGs [28], and two co-assembled SAGs of RS1 (Medium quality) and RS2 (High-quality) were obtained, which were not described in the previously reported catalogue of microbial draft genomes from the Red Sea [29]. Both were >2.8 Mb in genome size with >2900 coding DNA sequences (CDSs) and shared high sequence identities of 5S rRNA (109 bp) and 23S rRNA (2558 bp) (Table S5). In the NCBI taxonomy database, 143 draft genomes of 20 species including uncultured *Rhodobacter* have been registered. Analysis of 5S, 16S, and 23S rRNA sequence identity showed that the most closely related species to RS1 and RS2 was the *Rhodobacteraceae* bacterium HIMB11 isolated from coastal seawater in the Kaneohe Bay [30], exhibiting >99.6% identity. When the genome completeness of RS1 and RS2 was considered, estimated genome sizes and the number of CDSs (3.1 Mbp and 3183 in HIMB11, respectively) were comparable to each other. However, genome alignment revealed that 86.81% and 70.04% of the RS1 and RS2 genome sequences respectively exhibited ≤75% nucleotide identity to that of HIMB11 (Fig. S3B, C). ANI of the whole genome was 91.7% (RS1–RS2), 91.4% (RS1–HIMB11), and 92.4% (RS2–HIMB11).

**Fig. 4:**
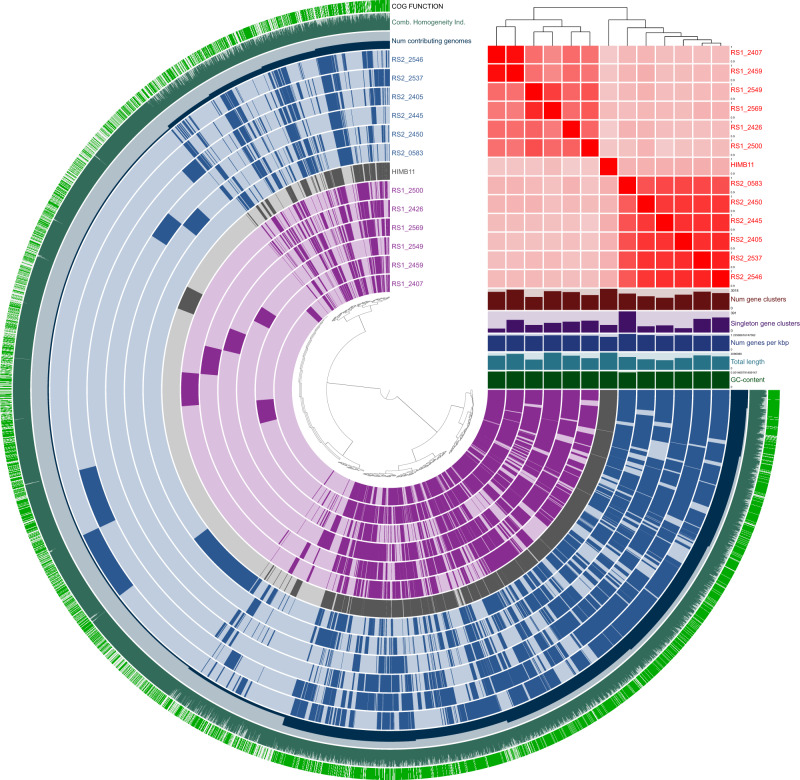
Comparative genome analysis of *Rhodobacter* spp. SAGs obtained from the Red Sea seawater. Anvi’o representation of *Rhodobacter* spp., including six RS1 SAGs (Purple) and six RS2 SAGs (Navy Blue) recovered from seawater. HIMB11 (Gray), the reference genome, is the genome of known *Rhodobacter* sp. containing >99% identical 16S rRNA gene. The heat map in the upper right part shows the ANI values of RS1, RS2, and the reference genome, and the details are shown in Fig. S4. The figure shows the presence/absence of 6274 gene clusters in the pangenome of 12 SAGs and the reference genome. Inter-strain average nucleotide identity was <94%, and RS1-specific or RS2-specific gene clusters were detected.

We compared the CDSs of SAGs, six from RS1 and six from RS2, with the reference genome of HIMB11, showing that each strain shared unique gene sets (Fig. 4). In total, 34,423 genes classified into 6274 clusters were identified. Only 6.9% (2372/34423) were found in one SAG, of which 92.8% (2202/2372) could not be annotated. KEGG pathway analysis revealed that the module completion ratio (MCR) of the glucose/mannose transport system and fructose transport system was 25% in RS1 and RS2, while that of the phosphonate transport system, iron complex transport system, zinc transport system, putative zinc/manganese transport system, and lipopolysaccharide export system was 100% in RS1 and RS2 (Table S6).

### Characteristic gene distributions in different sampling sites at single-cell resolution

**Fig. 5:**
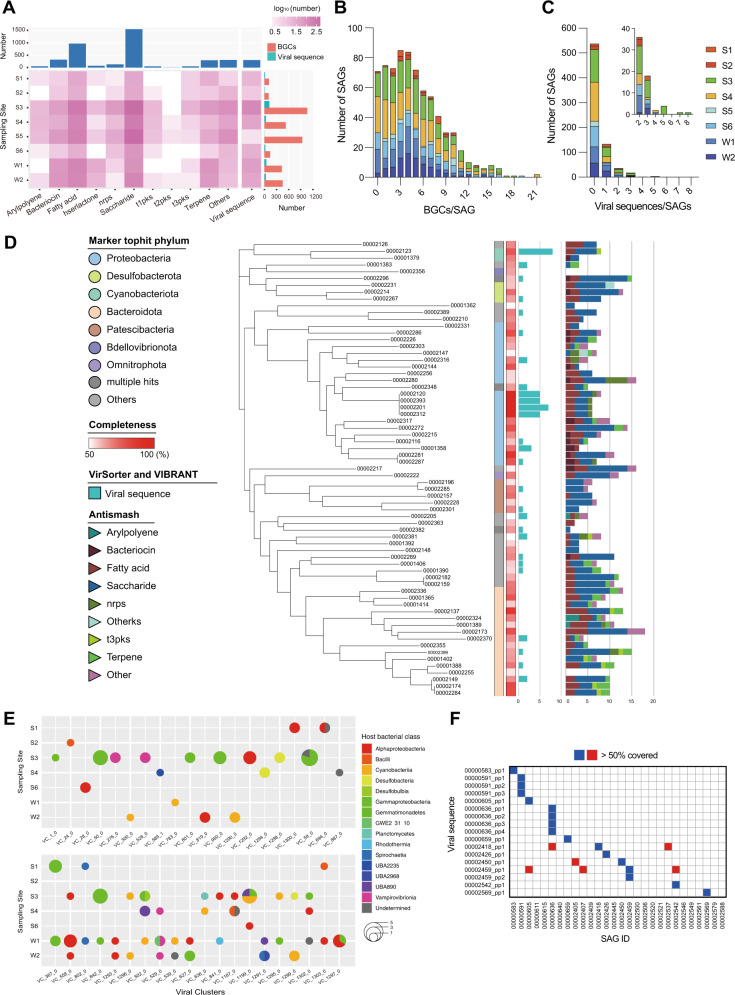
Comprehensive analysis of bacterial SAGs reveals the distributions of secondary metabolite biosynthetic gene clusters (BGCs) and viral signals. **A** The heat map and bar plots show the number and category of BGCs classified and viral sequences detected from each SAG. BGC category with the numbers <10 were classified as “others”. BGCs assigned as “cf_putative” were not counted. **B** Distributions of the BGC number per SAG. **C** Distributions of the viral sequence number per SAG. **D** Phylogenic tree with taxonomical classification, SAG completeness, and the number of viral sequences and BGCs. High-to-medium quality SAGs in S3: mangrove soil are shown. **E** Bubble pie chart summarizes 40 viral clusters (VCs) containing 177 viral sequences from sampling sites and host bacterial classes. The upper chart shows 20 VCs detected from a single bacterial strain (including “Undetermined”) at a single sampling site. In the lower chart, the 19 VCs from the left represent VCs detected from multiple sampling sites, and the 14 VCs from the right represent VCs detected from multiple bacterial classes. Viral sequences that were determined to be “Undetermined” at the class level but were determined to be a different phylum from sequences in the same VC are included in the lower chart. **F** Viral sequence profiling of *Rhodobacter* SAGs from the Red Sea seawater. The common presence of viral sequences was searched from *Rhodobacter* SAGs by mapping raw sequence reads. The blue squares represent viral sequences detected from the original SAG, and the red squares represent viral sequences detected from a different SAG from the original one.

Characteristic genes within 734 SAGs were searched, including BGCs and viral sequences. In total, 3676 BGCs were detected, with about 94.6% (694/734) of SAGs carrying at least one BGC (Table S7). Saccharide was dominant (41.5%), followed by fatty acid-related BGCs and bacteriocin-related BGCs (Fig. 5A). While the median number of BGCs in single cells was 5.0, two SAGs assigned as the closest species of Acidobacteria bacterium UBA890 and Oligoflexales bacterium from sea sediment (S4) contained 21 BGCs (Fig. 5B).

Next, we searched for viral sequences in SAGs, including sequences of proviruses in the bacterial genome, viruses with a lytic cycle, and some viruses attached to the bacterial cell membrane. From 196 SAGs, a total of 303 viral sequences were detected (Table S8). Mangrove soil (S3) showed the highest number of 124 sequences, followed by harbor seawater (W1) and fresh sea sediment (S4) (Fig. 5A). Of these, 74 (24.4%) were classified as integrated proviruses using VirSorter [31], and 71 (23.4%) were estimated to be temperate viruses using BACPHLIP [32]. The viral host lineage at the family level could be determined for about 76.6% (232/303) of the viral sequences. For example, a SAG from mangrove samples (S3) assigned as the closest species of the Cyanobacteria bacterium UBA9579 carried eight viral sequences (Fig. 5C), although some viral sequences were detected as duplicates of fragmented sequences. As shown in Fig. 5D, single-cell-resolved genome comparison revealed the number of BGCs and traces of viral infections in 70 medium- or high-quality SAGs collected from mangrove soils (S3). The results for the 260 medium- or high- quality SAGs collected from all sampling sites are shown in Fig. S4.

Following the criteria of Minimum Information about an Uncultivated Virus Genome [33], we searched 303 viral sequences using IMG/VR v3 [34] and found only 26 sequences. In addition, only a few sequences were found in the previously reported metagenome assembly from the Red Sea [35] and in the raw sequencing data (122 Gb) of a previous Red Sea metagenome study [36]. Network protein clustering of viral sequences revealed 40 viral clusters (VCs) (Table S8), which could be linked to the host bacterial classes and sampling sites (Fig. S5A, B). Only nine viral sequences formed clusters with the database, suggesting that most of the viral sequences are novel at the genus-subfamily level. The geographic distribution and the host range of viral sequences were assessed based on the results of VC clustering, and 20 VCs were detected from a single bacterial class in a single environment. Meanwhile, among the remaining 20 VCs, 19 VCs were detected from multiple sampling sites and 14 VCs were from multiple host bacterial classes (Fig. 5E).

To elucidate the function of these viral sequences in host bacteria, potential auxiliary metabolic genes (AMGs) were searched, and 95 AMGs were detected from 52 viral sequences, which included genes associated with ribonucleotide reductase (PF02867), photosynthetic reaction centre proteins (PF00124), and DNA methylase (PF00145) (Table S9). We also analyzed the viral sequence profiles of the 28 *Rhodobacter* spp. SAGs analyzed above. Although 17 viral sequences were detected from the 28 SAGs, only some viral sequences were common among the multiple SAGs in the raw read sequence data (Fig. 5F), and none of them was commonly detected in contigs.

## Discussion

The current single-cell genome sequencing usually involves the isolation of single cells by FACS [37], followed by cell lysis and WGA in individual wells. In this process, the lysis methods that can be applied are limited, because the reagents for cell lysis should be carried over to the WGA. On the other hand, with the SAG-gel developed herein, single cells and DNA are captured in an agarose matrix, enabling comprehensive lysis by combining multiple lysis methods through repeated washings of gel beads. Validation using model microbes confirmed a 9.1–25% increase in genome coverage, indicating that efficient WGA could be achieved (Fig. 1).

In addition, FACS-based cell sorting faces some difficulties when applying to various environmental samples. Especially in samples containing complex particles such as soils, background noise affects sorting efficiency and results in a low throughput of WGA [19]. SAG-gel also uses FACS, but the isolation of gel beads harboring amplified DNA, rather than cells, can improve the sorting efficiency, because the fluorescence signal from the gel beads is sufficiently large compared to the noise from particles. We also confirmed that the co-encapsulation of particles had no effects on WGA by evaluating the fluorescence-positive rate of gel beads and sequence quality.

SAG-gel could be applied to diverse environmental samples, including those from soil seawater, without protocol modification, and >35% of the SAGs were classified as high-to-medium quality (Fig. 2). SAG-gel also yielded 57.8% of SAGs containing almost full-length 16S rRNA genes from the complex microbial communities, outperforming conventional single-cell genomics (33.8% and 24.9%) [38, 39] and metagenomic binning. The combination of the randomized SAG-gel pool and FACS-based isolation revealed a variety of bacterial species that have not been described from soil and seawater (Fig. 3). The SAG-gel approach has shown the ability to enhance the reference genome diversity by recovering genomes from uncultured environmental bacteria.

The current metagenomic binning approach is powerful enough to estimate environment-specific gene contents and produce draft genomes based on nucleotide composition and coverage depth. However, when multiple species or strains belonging to the same genera are present at similar abundances and have similar nucleotide compositions, the metagenomic binning approach often fails to separate contigs from these taxa into the correct genome bins [8], and the 16S rRNA gene recovery rate is low for the same reasons [23]. On the other hand, our approach enabled us to acquire detailed genome sequences from individual cells, thereby revealing the co-existence of multiple *Rhodobacter* strains harboring highly conserved 16S rRNA gene sequences but having different genome contents (Fig. 4 and Fig. S3). The presence of bacteria that are taxonomically identical but have different profiles of functional modules is potentially important for understanding the functional diversities of ecosystems, especially in the sequencing of geographically separated environmental samples and symbiotic or commensal bacteria from different hosts.

Single-cell genome sequencing with SAG-gel also enabled the detection of characteristic genes from individual SAGs, potentially contributing to the accumulation of novel BCGs and viral sequences (Fig. 5). As in metagenomic analysis, it was difficult to obtain full-length BGCs from short read assemblies, while >2900 BGCs larger than 10 kbp were detected from SAGs, indicating that BGCs could be collected with comparable or higher efficiency compared with previous metagenomic analyses [40]. Likewise, while some viral sequences were also fragmented, SAG-gel was able to link 76.6% of the viral sequences to host lineages, which is more robust and comprehensive than the conventional metagenome-based approach (7.7% and 14.4%) [9, 41]. We found that 24.4% of viral sequences were integrated in SAGs, which was slightly higher than the result of a recent incubation experiment with planktonic viruses from the coastal Red Sea [42]. Since most of the free viral particles are removed by the filtration and washing steps during sample preparation, the possibility of mispacking is considered to be low.

Combining the information of host bacterial taxon, half of the VCs detected were distributed across multiple bacterial classes or sampling sites (Fig. 5E). Previous studies have shown that virus genera are specific for host families [43], while the sequences within the same VC are assumed to be related at the genus-subfamily level [44], suggesting that the VCs detected from multiple bacterial classes possibly have diverse host ranges. Since about 86% of the viral sequences have not been described in the current database, further verifications, such as virus-engineering with synthetic biology [45] and binding domain simulations [46], are required to evaluate whether these widely distributed VCs are characteristic to the Red Sea and nearby area or prevalent worldwide. In addition to the diverse host range of viruses, viral sequence profiling of *Rhodobacter* spp. SAGs implied that random infection of diverse viruses increases the diversity of host bacterial genomes at an individual scale (Fig. 5F). Further accumulation of viral sequences will promote our understanding of viral functions including bacterial population control [47] and of horizontal gene transfer [48].

In this study, we demonstrated that extensive single-cell genome sequencing using SAG-gel links bacterial taxonomy to metabolic functions, thus enabling the search for target genes in bacterial communities. SAG-gel is a platform for high-throughput and accurate single-cell whole genome sequencing for a variety of environmental samples, including soil and seawater. Future applications for deep and large-scale genome sequencing will not only facilitate the elucidation of microbial dark matter, but will also be useful in analyzing gene transfer and virus-host interactions in the complex microbial community.

## Materials and methods

### Single-cell genome sequencing of model bacteria with SAG-gel

Lab-cultured *E. coli* K-12 strain (ATCC 10798; genome size: 4.6 Mbp) and *B. subtilis* (ATCC 6633; genome size: 4.0 Mbp) were used as model bacteria. Detailed protocols about preparation of bacterial fractions, WGA in agarose gel beads, sequence library construction, and sequencing have been described in the Supplementary Information.

### Preparation of bacterial fractions from environmental samples

Six soil samples [beach soil: S1 (22°17’35.6”N 39°05’26.0”E); desert soil: S2 (22°19’03.0”N 39°08’36.7”E); mangrove soil: S3 (22°18’53.3”N 39°05’29.2”E); fresh sea sediment: S4 (22° 17.988’N, 39° 03.427’E); frozen sea sediment: S5 (22° 17.988’N, 39° 03.427’E); and seashore soil: S6 (22°17’17.5”N 39°05’42.3”E)] and two seawater samples [harbor seawater: W1 (22°18’16.9”N 39°06’12.3”E) and open-ocean seawater: W2 (22° 17.988’N, 39° 03.427’E)] were collected from the Red Sea and the nearby area. For soil samples, ten-gram samples were used to prepare the bacterial fractions. For seawater samples, four-liter samples were used. The bacterial fraction of soil was fractionated by centrifugation after suspension in Dulbecco’s phosphate-buffered saline (−) (DPBS, Thermo Fisher Scientific) and that of seawater was prepared by suction filtration. Each bacterial fraction was centrifugally washed three times in DPBS and proceeded to the following steps. Detailed protocols on sampling and preparation of cell suspensions are provided in the Supplementary Information.

### Single-cell encapsulation into agarose gel beads

A microfluidic device with cross junction was fabricated as previously reported [49] for generating monodispersed picoliter-sized droplets. Ultra-low gelling temperature agarose A5030 (Sigma-Aldrich) was mixed with DPBS (3% w/v) and incubated at 85 °C for 30 min. The cell concentration was calculated with bacterial counter after SYBR Green I (S7563, Thermo Fisher Scientific) staining, and mixed with agarose solutions to prepare 1.5% w/v agarose cell suspensions with 3.0 × 10^3^ cells/μL. Agarose cell suspensions were loaded into a PTFE tube (AWG 24) connected to a Mitos P-pump (Dolomite, Charleston, MA, USA) and introduced into a microfluidic device with 2% Pico-Surf1 in Novec7500 (Dolomite) as carrier oil. Microfluidic droplets with a diameter of 40 μm (volume: 34 pL) were generated for encapsulation of >100,000 single cells within 30 min (35,000 droplets/min) at a concentration of 0.1 cell/droplet. Droplets were collected in 1.5 mL tubes from the outlet, and incubated on ice for 15 min, and broken by 1H,1H,2H,2H-perfluoro-1-octanol (Sigma-Aldrich). Gel beads were washed sequentially with 500 μL of acetone (Sigma-Aldrich), isopropanol (Sigma-Aldrich), and DPBS (DPBS for three times).

### Cell lysis and 1st-round WGA in agarose gel beads

Gel beads were subjected to enzyme cocktail treatment. Two hundred microliters of lysozyme solution (50 U/μL Ready-lyse lysozyme [Lucigen, WI, USA], 2 U/mL zymolyase [Zymo Research, Orange, CA, USA], 22 U/mL lysostaphin [Sigma-Aldrich], and 250 U/mL mutanolysin [Sigma-Aldrich] in DPBS) was added and incubated at 37 °C overnight. Gel beads were washed with DPBS three times and 200 μL of achromopeptidase solution (0.5 mg/mL achromopeptidase [Wako, Tokyo, Japan] in DPBS) was added and incubated at 37 °C for 8 h. The washing steps were repeated, and 200 μL of proteinase K solution (1 mg/mL proteinase K [Promega, Madison, WI] and 0.5% SDS [Wako, Tokyo, Japan] in DPBS) was added and incubated at 40 °C overnight. After repeating the washing steps five times, 4 μL of gel beads suspension was dispensed into 0.2 mL tubes (Axygen Biosciences) and 3 μL of D2 buffer from a REPLI-g Single Cell Kit (QIAGEN) was added. After incubation at 40 °C for 10 min, 43 μL of WGA mixture (3 μL of Stop Solution, 9 μL of H_2_O, 29 μL of reaction buffer, and 2 μL of DNA polymerase) was added and incubated at 30 °C for 3 h.

### Confirmation of DNA amplification and isolation of gel beads

Gel beads were washed with DPBS three times, stained with 1× SYBR Green (Thermo Fisher Scientific) in DPBS, and transferred onto a glass slide for microscopic observation. Fluorescence-positive gel beads were isolated by FACS or manual picking. Gel beads from S1, S2, S5, S6, and W1 were subjected to manual picking within 2 days after WGA. The remaining samples were isolated by FACS within 1 week. Both manual picking and FACS-based isolation were conducted for S6 and W1. From the eight sampling sites, 48–480 fluorescence-positive gel beads were isolated and subjected to 2nd-round WGA or maintained at –30 °C for long-term storage.

1. **Single-droplet sorting with manual picking**
  Fluorescence-positive gel beads were manually picked using a micropipette (Drummond, Camlab, Cambridge, UK) under the open clean system (KOKEN LTD.). Each bead was dispensed into a 96-well plate with 0.8 μL of DPBS.
2. **Single-droplet sorting with FACS**

Fluorescence-positive gel beads were sorted into 96-well plate using a BD FACSMelody^™^ Cell Sorter (BD Biosciences) equipped with a 488 nm excitation laser. The nozzle diameter was 100 μm. The sample flow rate was adjusted to ~30 events per second. The number of sorted beads is summarized in Table S1 and S3. Prior to gel-bead sorting, 0.8 μL of DPBS was dispensed into each well. Fluorescence-negative gel beads were also sorted into another 96-well plate (at least *n* = 3 for each environment sample) for evaluating background amplification.

### 2nd-round WGA

We added 0.6 μL of D2 buffer to each well and incubated at 65 °C for 10 min. Then 8.6 μL of WGA mixture (0.6 μL of Stop Solution, 1.8 μL of H_2_O, 5.8 μL of reaction buffer, and 0.4 μL of DNA polymerase) was added and incubated at 30 °C for 2 h. The WGA reaction was terminated by heating at 65 °C for 3 min, and master library plates of SAGs were prepared.

### DNA quantification and 16S rRNA gene PCR

The amplicon yields of the 2nd-round WGA were quantified using a Qubit dsDNA HS assay kit (Thermo Fisher Scientific). To estimate the samples derived from bacteria, 16S rRNA gene PCR and agarose electrophoresis (100 V, 15 min) was performed against the WGA amplicons. Primer pair sequences for the V3V4 region were used according to Illumina’s MiSeq system protocols (Forward: 5′-TCGTCGGCAGCGTCAGATGTGTATAAGAGACAGCCTACGGGNGGCWGCAG-3′, Reverse: 5′-GTCTCGTGGGCTCGGAGATGTGTATAAGAGACAGGACTACHVGGGTATCTAATCC-3′).

### Construction of single-cell genome libraries and whole-genome sequencing

Illumina libraries were prepared using the 2nd-round WGA amplicons with Nextera XT DNA sample prep kit (Illumina) according to the manufacturer’s instructions. Libraries were sequenced on an Illumina HiSeq for 150 cycles of paired-end sequencing (GENEWIZ, South Plainfield, NJ, USA), generating a total of 356 Gbp (1.19 billion paired-end reads).

### De novo assembling and phylogenetic analysis

Sequence reads were de novo assembled with SPAdes (v3.12.0, options: -sc –careful) [50], and contigs (>1000 bp) underwent the following analysis. Completeness and contamination were calculated with CheckM (v1.1.0, lineage_wf) [51]. The number of contigs, N50, and total length were evaluated with QUAST v4.5 [52]. The number of CDS, rRNAs, tRNAs was examined with Prokka v1.12 [53]. The SAG quality was evaluated by the standards developed by the Genomic Standards Consortium [22]. After removing samples that were classified as contaminants or exhibited 0% of completeness, the remaining SAGs underwent the following analysis: 16S rRNA gene sequence was extracted from the Prokka result [53] and assigned to the 16S rRNA gene sequence database (Silva 132). The 16S rRNA genes which exhibited <97% identity to the top hit result to the database were denoted to have no obvious reference sequences. SAGs were taxonomically classified with GTDB-Tk (v1.0.2, classify_wf) [54] and were visualized with iTOl [55]. The GTDB-Tk results were summarized at the phylum level to compare the bacterial composition of SAGs and 16S rRNA gene sequencing. We evaluated the number of species using the Mash distance 0.05 as the cutoff for species delineation (Mash v2.0) [56].

### Genome analysis of *Rhodobacter* spp

SAGs that exhibited >95% ANI were co-assembled with ccSAG [28], and co-assembled SAGs and the draft genome of *Rhodobacteraceae* bacterium HIMB11(GCF_000472185) were evaluated with QUAST [52], CheckM [51], Prokka [53], FastANI v1.3 [57], and Anvi’o v5 [58]. Co-assembled SAGs of RS1 and RS2 were aligned to the shotgun sequencing data of HIMB11 with D-GENIES v1.2.0 [59]. Each co-assembled SAG was searched against Genomaple-2.3.2 [60], and MCR in each functional module was evaluated.

### Statistical analysis for each SAG

SAGs were referenced against antiSMASH v4.2.0 [61] (search option: -transatpks_da –clusterblast –subclusterblast –knownclusterblast –smcogs –inclusive –borderpredict –full-hmmer), VirSorter v1.0.6 [31], VIBRANT v1.2.1 [62], and Plasmidfinder v2.1 [63] for detecting BGCs, viral sequences, and plasmids, respectively. For antiSMASH, BGCs of detected numbers <10 were grouped as “others”. The sequences detected in both VirSorter and VIBRANT were assigned as viral sequences. Whether the viral sequences were temperate or virulent was investigated by VirSorter and BACPHLIP v0.9.6 [32]. The viral sequences were evaluated with CheckV [64] and searched for IMG/VR3 (October 2020 version) [34] with blastn to determine the number of new viruses acquired at the vOTU level. We used thresholds of vOTU as 95% ANI over 85% alignment fraction according to Minimum Information about an Uncultivated Virus Genome [33]. Furthermore, the viral sequences were also searched for the virome data [35] or the raw sequencing data from the past Red Sea metagenomes [36] by mapping the sequence reads by bwa mem(0.7.10) [65]. Threshold of the mapping detection was set to 75% coverage.

We used vConTACT2 v0.9.20 to cluster the viral sequences via the Infrastructure for a PHAge Reference Database perl script (accessed on 1 November 2021) (-rel-mode ‘Diamond’ –db ‘None’ –pcs-mode MCL –vcs-mode ClusterONE) [66]. The network diagram of vConTACT2 [67] was visualized using Cytoscape v3.8.2 [68]. AMGs in viral sequences were predicted using DRAM-v v1.2.4 [69]. All 303 viral sequences were searched against the database without KEGG and UniRef using DRAM-v without an output file of the newer version VirSorter. We retained the genes flagged as “metabolic” as putative AMGs. Synteny analysis of genomic region was performed using Clinker version 0.0.23 [70]. The 17 viral sequences from *Rhodobacter* spp. SAGs were searched for raw sequencing data in this study by bwa mem (0.7.10) [65]. Considering the completeness of *Rhodobacter* spp. SAGs, the mapping detection threshold was set to 50% coverage.

## Supplementary information

Supplementary information

Figure S1

Figure S2

Figure S3

Figure S4

Figure S5

Table S1

Table S2

Table S3

Table S4

Table S5

Table S6

Table S7

Table S8

Table S9

## Data Availability

Sequence raw data of 929 SAGs are available in the DDBJ Sequence Read Archive under the accession numbers DRA009438- DRA009443 (Table S4). Sequences of 303 viral sequences are also available under the accession numbers LC662857-LC663159 (Table S8). If you need more information, please contact the corresponding author.
