# Supplementary material for: Validation of the application of gel beads-based single-cell genome sequencing platform to soil and seawater
Source: ISME Commun. 2022 Sep 29;2:92. doi: 10.1038/s43705-022-00179-4 (PMC9723564; doi:10.1038/s43705-022-00179-4)
Supplement: Supplementary file 1 — Supplementary information [file 43705_2022_179_MOESM1_ESM.docx]

**Supplementary Information**

**Preparation of bacterial fractions of model bacteria**

For genome sequencing analysis, *E. coli* K-12 strain (ATCC 10798; genome size: 4.6 Mbp) and *B. subtilis* (ATCC 6633; genome size: 4.0 Mbp) were used as model bacteria. *E. coli* K-12 cells were pre-cultured in Luria-Bertani (LB) medium (1.0% Bacto tryptone [BD Biosciences, Franklin Lakes, NJ, USA], 0.5% yeast extract [BD Biosciences], 1.0% NaCl [Sigma-Aldrich, Hamburg, Germany], pH 7.0) for 16 h. *B. subtilis* cells were pre-cultured in brain heart infusion broth (ATCC medium 44; Thermo Fisher Scientific, Waltham, MA, USA.) for 16 h. For cell collection, 1 mL of cultured medium was dispensed into a 1.5 mL tube and centrifuged at 8000 × g for 5 min. After removing the supernatant, the collected cells were resuspended in UV-treated Dulbecco’s phosphate-buffered saline (-) (DPBS, Thermo Fisher Scientific) and washed three times with DPBS. Finally, the cells were resuspended in 500 μL DPBS and cell concentration was estimated using a bacterium counter. To evaluate cross-contamination, *E. coli* and *B. subtilis* cells were mixed at a ratio of 1:1. For single-cell encapsulation, the cell concentrations of *E. coli* and *B. subtilis* were adjusted to 3.0 × 10^3^ cells/μL at a concentration of 0.1 cell/droplet. All preparations for the cell suspension and further processes were performed under an open-interior clean bench (KOACH T 500-F; KOKEN LTD., Tokyo, Japan) except for droplet generation and isolation with FACS.

**Cell lysis and WGA in agarose gel beads**

After the gel beads were collected with a tabletop centrifuge and supernatant was removed, they were treated with two different lysis protocols: Alkaline treatment and Enzyme cocktail treatment.

1. **Alkaline treatment and WGA**

The following steps were performed in 0.2 mL tubes (Axygen Biosciences): 3 μL of D2 buffer from a REPLI-g Single Cell Kit (QIAGEN) was added to 4 μL of droplet suspension. After incubation at 40℃ for 10 min, 43 μL of WGA mixture (3 μL of Stop Solution, 9 μL of H2O, 29 μL of reaction buffer, and 2 μL of DNA polymerase) was added and incubated at 30℃ for 3 h.

1. **Enzyme cocktail treatment and WGA**

The enzyme cocktail treatment was performed by the same protocol described in the main manuscript.

**Construction of single-cell genome libraries and whole genome sequencing of model bacteria**

After confirmation of DNA amplification with 1× SYBR Green (Thermo Fisher Scientific), fluorescence-positive gel beads were isolated by FACS. Subsequently, 2^nd^-round WGA was performed using the REPLI-g Single Cell Kit (QIAGEN). Illumina libraries were prepared using amplicons from the 2^nd^-round WGA products using a Nextera XT DNA sample prep kit (Illumina) according to the manufacturer’s instructions. Libraries were sequenced on an Illumina MiSeq for 75 cycles of paired-end sequencing, generating a total of 50.0 million paired-end reads and 7.34 Gbp.

**Sequencing analysis for model bacteria**

The sequence raw reads were mapped to the NCBI reference genome (NIH, Bethesda, MD, USA) with BWA(1) for evaluation of cross contamination. NC_00913 (*E. coli* substrain MG1655) and CP011496 (*E. coli* strain NCM3722 plasmid F) were used as a reference for the *E. coli* K-12 strain. NCBI reference genome NC_014479 was used for *B. subtilis* subsp. spizizenii str. W23. Then the acquired reads were down sampled to 10× mean mapping depth (46 Mbp for *E. coli* and 40 Mbp for *B. subtilis*). Sequence reads were *de novo* assembled with SPAdes 3.5.0 (2) and qualified by QUAST 2.3 (3). Contigs (> 500 bp) from each sample were mapped to the reference genome with BWA. Genome coverage was calculated using SAMtools (4).

**Proof of SAG-gel concept with *E. coli* and *B. subtilis***

When we encapsulated *E. coli* at a concentration of 0.1 cell/droplet (i.e., one cell per 10 droplets), the rate of fluorescence-positive beads after WGA was 10.5%, suggesting that single cells successfully reacted with the lysis buffer and WGA mixture. In addition, when we generated blank beads that contain no cells, the rate of false-positive beads was less than 0.05%. Hence, we adjusted the cell concentration to 0.1 cell/droplet (3000 cells/μL) to prevent the co-encapsulation of multiple cells in gel beads (0.47% for co-encapsulation).

The use of enzyme cocktails enhanced the genome coverage of *E. coli* and *B. subtilis* by 9.1–25% (Fig.1D). As indicated in our previous report (5), *B. subtilis* showed superior coverage performance though there were several outliers, which may be attributed to the lower GC content and genome size of *B. subtilis* (43.9% and 4.0 Mbp) compared with those of *E. coli* (50.8% and 4.6 Mbp). Several outliers may be attributed to apoptotic cells, cellular debris, or incompletely lysed cells. The cells were embedded in an agarose matrix containing numerous pores, allowing the access of lysis enzymes and detergents while preventing DNA from physical shearing, which improves the accessibility of phi29 polymerase to single-cell DNA and the amplification efficiency. In addition, SAG-gel excludes the risk of cross-contamination between gel beads, showing that all the sequenced SAGs had > 99.6% of their reads mapping to either *E. coli* or *B. subtilis* (Fig. 1F). This result outperformed the previously reported single droplet MDA (sd-MDA) (5), suggesting that contaminated short DNA fragments were removed by washing steps. We also identified the attribution of unmapped reads, which were mainly ascribed to *Homo sapiens*, *Propionibacterium*, and *Pseudomonas*, which are often observed as laboratory contaminants (6). The number of unmapped reads were equivalent to the results from purified extracted genomic DNA (gDNA), suggesting that DNA contamination in SAG-gel is not derived from WGA.

In SAG-gel platform, the SAG-containing beads can be stored for months at 4℃ and the plate-based SAG library can be frozen for longer storage, expanding the possibility of performing scaled-up sequencing in various sequencing machines on demand.

**Preparation of bacterial suspensions from environmental samples**

Six soil samples [beach soil: S1 (22°17’35.6”N 39°05’26.0”E); desert soil: S2 (22°19’03.0”N 39°08’36.7”E); mangrove soil: S3 (22°18’53.3”N 39°05’29.2”E); fresh sea sediment: S4 (22° 17.988’N, 39° 03.427 ’E); frozen sea sediment: S5 (22° 17.988’N, 39° 03.427 ’E); and seashore soil: S6 (22°17’17.5”N 39°05’42.3”E)] and two seawater samples [harbor seawater: W1 (22°18’16.9”N 39°06’12.3”E) and open ocean seawater: W2 (22° 17.988’N, 39° 03.427 ’E)] were collected. For S1, S2, S3, and S6 samples, 20–30 g of soil was collected 10 cm beneath the top layer and maintained in 50 mL tubes on ice until preparation of the cell suspension. For S4 and S5 samples, 20–30 g of soil was collected from the seafloor at a depth of 25 m with van Veen Grab sampler and kept into 50 mL tubes. For the S5 sample, sea sediment was collected on 30^th^ April 2017, frozen by dry ice, and directly kept on -80℃ for 14 months. It was thawed on ice immediately before the preparation of cell suspensions. S4 was also kept on ice until the preparation of cell suspensions. W1 and W2 were collected from the marine surface. From each sampling site, 30 L of seawater was collected into plastic tanks. Cell suspensions were prepared as soon as possible after arrival at the laboratory. All samples except for S5 were collected from the 24^th^ to the 27^th^ of June 2018 and proceeded to WGA. Ten grams each of samples S1 to S6 was dispensed into three 50 mL tubes (Iwaki Science Products Department, Iwaki Glass Co. Ltd., Chiba, Japan). DPBS was added to each tube up to the 40-mL volume marker and the contents were mixed thoroughly. The suspended solution was kept on ice for 5 min and the supernatant was collected into another 50 mL tube. The supernatant was filtered with a 5-μm MF-Millipore membrane filter (Merck Millipore, Milan, Italy). The flow-through was centrifuged at 10,000 ×g for 5 min with a benchtop centrifuge (75004251, Thermo Fisher Scientific). After removing the supernatant, the pellet was resuspended in 10 mL of DPBS and dispensed into 1.5 mL tubes (Axygen Biosciences, Hangzhou, China). Each suspension was centrifuged at 10000 ×g for 5 min with a tabletop ultracentrifuge and washed three times with DPBS. For the seawater samples, 4 L of seawater was filtered with a 5-μm MF-Millipore membrane filter. The flow-through was collected and filtered with a 0.22-μm filter (Merck Millipore). Then the 0.22-μm filter was suspended in 10 mL of DPBS and vortexed thoroughly to suspend trapped bacterial fractions; 10 mL of bacterial suspensions was dispensed into 1.5 mL tubes, centrifuged at 10000 ×g for 5 min with a tabletop ultracentrifuge, and washed three times with DPBS. We prepared two tubes of cell suspensions for each sample. One tube was used for single-cell genome sequencing and the other for 16S rRNA gene sequencing. For single-cell genome sequencing, the cell concentration was calculated with bacterial counter after SYBR Green I (S7563, Thermo Fisher Scientific) staining, and adjusted to 3.0 × 10^3^ cells/μL for encapsulating single cells at a concentration of 0.1 cell/droplet (40 μm diameter). For 16S rRNA gene sequencing, total metagenomic DNA was extracted with PowerLyzer Soil DNA Extraction kit (QIAGEN, Hilden, Germany) from the cell suspensions. Then 16S rRNA gene sequencing libraries were prepared according to Illumina’s protocol and run on an Illumina MiSeq for 300 cycles of paired-end sequencing using a MiSeq v3 600-cycle reagent kit (Illumina, San Diego, CA, USA). For S1, the amount of DNA extracted was insufficient to prepare the sequencing library.

**Comparison of bacterial phylogeny acquired by SAG-gel and 16S rRNA gene sequencing**

We compared the bacterial taxonomic composition at the phylum level obtained from SAGs with that of metagenomic 16S rRNA gene amplicon sequencing. While the number of major phyla (> 5% in relative abundance) detected in 16S rRNA gene sequencing was 11, SAGs recovered 10 phyla—all except for *Chloroflexi*, which is reported to be a tough bacterium for DNA extraction (7). The bacterial composition of SAGs was not consistent with that of 16S rRNA gene sequencing except for mangrove (S3), where the result also corresponded to the previously reported data (8) (Fig. S3A, B). In 16S rRNA gene sequencing, the bacterial composition is affected by copy number variation of 16S rRNA gene and amplification bias even if some correction tools are used (9, 10). On the other hand, in single-cell genome analysis, the bacterial composition is affected by differences in cell lysis efficiency and insufficient number of analyzed cells. As the principle of each technique and its applicability are different, the bacterial composition of SAGs did not agree well with the result from 16S rRNA gene sequencing. Although there was a bias in the bacterial composition, SAG-gel could recover very rare phyla (< 0.1% in 16S rRNA gene sequencing), including *Omnitrophota* bacteria and *Elusimicrobiota* bacteria which have been proposed recently and classified as uncultured phyla (11, 12). SAG-gel could also recover three high-quality and ten medium-quality SAGs of SAR11 clade bacteria, which is difficult to be subject to genome assembly by metagenomics due to their large diversities and population sizes (13).

**Fig. S1. Completeness and contamination statistics for SAGs obtained from eight environmental samples.**

Genome completeness and contamination of each SAG are plotted by each sampling site. S6 and W1 were processed using both manual beads picking and FACS-based beads isolation. Samples which were classified as contamination and exhibited 0% completeness were also included.

**Fig. S2. Microbial composition in each sampling site and data collection method (16S: 16S rRNA gene sequencing; SAG: SAG-gel).**

(A) Comparison of bacterial composition in 16S rRNA gene sequencing and SAGs (Phylum level). Taxonomic identification of SAGs was performed using GTDB-Tk. In S1, the bacterial composition of 16S rRNA gene sequencing was undetermined because of the shortage of extract DNA. Any phyla which shared < 5% was clustered as “others”. (B) Principal component analysis of microbial composition at phylum level.

**Fig. S3. Comparative genome analysis of *Rhodobacter* spp. SAGs obtained from harbor seawater (W1).**

(A) ANI of 28 SAGs classified as *Rhodobacter* spp. (B) (C) Sequence alignments of *Rhodobacter* spp. (B) RS1 and (C) RS2 against the shotgun sequencing data of HIMB11 with D-GENIES.

**Fig. S4. Summary of characteristics of SAGs classified as high-to-medium qualities.**

Genome completeness, taxonomical classification, the number of viral signals and metabolite biosynthetic gene clusters (BGCs) in all sampling sites were summarized for high- and medium- quality SAGs. In the result of BGCs, clusters assigned as “cf_putative” were not counted.

**Fig. S5. Taxonomic assignment and geographical distribution of the viral sequences.**

The 177 viral sequences acquired in this study were colored by (A) bacterial class of the SAG or (B) the sampling sites. Small gray dots represent reference viral sequences.

**Table S1. Number of gel beads processed to 2nd-round amplification, 16S rRNA gene PCR, and next-generation sequencing in model bacteria**

**Table S2. Summary of de novo assembling of *E. coli* genomes evaluated with QUAST**

SAG-gel was compared with conventional in-tube MDA and previously reported methods including compartmented droplet MDA (cd-MDA) (14), and single-droplet MDA (sd-MDA) (5).

**Table S3. Overview of SAG-gel sequencing for environmental samples**

Number of gel beads processed to 2nd-round amplification, 16S rRNA gene PCR, next-generation sequencing, and quality distributions are summarized for environmental samples.

**Table S4. Sequence statistics of 929 sequenced samples**

Summary of sequence statistics including accession number, number of contigs, total length, GC%, N50, completeness and contamination, kinds of tRNA, number and length of 5S, 16S, and 23S rRNA gene fragments, taxonomic annotation, 16S rRNA gene-based Blast search against the Silva database, Mash distance, refseq tophit, and p-value.

**Table S5. Sequence statistics of *Rhodobacter spp.* RS1 and RS2**

**Table S6. Summary of the results of genomaple (Complex module, Functional modules, Pathway modules, and Signature modules) on *Rhodobacter* spp. RS1 and RS2**

**Table S7. List of secondary metabolite biosynthetic gene clusters (BGCs)**

**Table S8. List of viral sequences**

Accession number and output results of VirSorter, vConTACT2, CheckV, BACPHLIP, and blastn are summarized.

**Table S9. List of potential auxiliary metabolic genes (AMGs) detected from 303 viral sequences with DRAM-v**

**Refferences**

(1) Li H, Durbin R (2009). Fast and accurate short read alignment with Burrows-Wheeler transform. *Bioinformatics* **25:** 1754-1760.

(2) Bankevich A, Nurk S, Antipov D, Gurevich AA, Dvorkin M, Kulikov AS *et al* (2012). SPAdes: a new genome assembly algorithm and its applications to single-cell sequencing. *J Comput Biol* **19:** 455-477.

(3) Gurevich A, Saveliev V, Vyahhi N, Tesler G (2013). QUAST: quality assessment tool for genome assemblies. *Bioinformatics* **29:** 1072-1075.

(4) Li H, Handsaker B, Wysoker A, Fennell T, Ruan J, Homer N *et al* (2009). The Sequence Alignment/Map format and SAMtools. *Bioinformatics* **25:** 2078-2079.

(5) Hosokawa M, Nishikawa Y, Kogawa M, Takeyama H (2017). Massively parallel whole genome amplification for single-cell sequencing using droplet microfluidics. *Sci Rep* **7:** 5199.

(6) Woyke T, Sczyrba A, Lee J, Rinke C, Tighe D, Clingenpeel S *et al* (2011). Decontamination of MDA reagents for single cell whole genome amplification. *PLoS One* **6:** e26161.

(7) Sutcliffe IC (2011). Cell envelope architecture in the Chloroflexi: a shifting frontline in a phylogenetic turf war. *Environ Microbiol* **13:** 279-282.

(8) Al-Amoudi S, Razali R, Essack M, Amini MS, Bougouffa S, Archer JA *et al* (2016). Metagenomics as a preliminary screen for antimicrobial bioprospecting. *Gene* **594:** 248-258.

(9) Kennedy K, Hall MW, Lynch MD, Moreno-Hagelsieb G, Neufeld JD (2014). Evaluating bias of illumina-based bacterial 16S rRNA gene profiles. *Appl Environ Microbiol* **80:** 5717-5722.

(10) Louca S, Doebeli M, Parfrey LW (2018). Correcting for 16S rRNA gene copy numbers in microbiome surveys remains an unsolved problem. *Microbiome* **6:** 41.

(11) Geissinger O, Herlemann DP, Morschel E, Maier UG, Brune A (2009). The ultramicrobacterium "Elusimicrobium minutum" gen. nov., sp. nov., the first cultivated representative of the termite group 1 phylum. *Appl Environ Microbiol* **75:** 2831-2840.

(12) Nobu MK, Narihiro T, Mei R, Kamagata Y, Lee PKH, Lee PH *et al* (2020). Catabolism and interactions of uncultured organisms shaped by eco-thermodynamics in methanogenic bioprocesses. *Microbiome* **8:** 111.

(13) Wilhelm LJ, Tripp HJ, Givan SA, Smith DP, Giovannoni SJ (2007). Natural variation in SAR11 marine bacterioplankton genomes inferred from metagenomic data. *Biol Direct* **2:** 27.

(14) Nishikawa Y, Hosokawa M, Maruyama T, Yamagishi K, Mori T, Takeyama H (2015). Monodisperse Picoliter Droplets for Low-Bias and Contamination-Free Reactions in Single-Cell Whole Genome Amplification. *PLoS One* **10:** e0138733.
